# Correction to “Human papillomavirus (HPV)‐18 E6 oncoprotein interferes with the epithelial cell polarity Par3 protein”

**DOI:** 10.1002/1878-0261.70278

**Published:** 2026-06-03

Facciuto F , Valdano MB , Marziali F , Massimi P , Banks L , Cavatorta AL , Gardiol D . Human papillomavirus (HPV)-18 E6 oncoprotein interferes with the epithelial cell polarity Par3 protein. Mol Oncol. 2014;8.10.1016/j.molonc.2014.01.002PMC552863924462519 https://doi.org/10.1016/j.molonc.2014.01.002.

In this article, an error occurred during figure assembly that resulted in the western blot images in Figure 4C being published with an incorrect tubulin control.

In the original version of Figure 4C, an unintentional error occurred involving both an inversion (flip) and an incorrect crop of the tubulin blot. Inspection of the original tubulin blot confirms that these errors did not confer any advantage to the interpretation of the data. The corrected figure has now been provided to accurately reflect the original results.

Figure 4C:

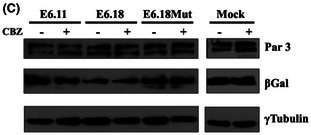

The authors agree to this corrigendum and confirm that these changes do not affect the conclusions of the article. The authors apologize for any inconvenience caused.

